# Comparison of contact angle hysteresis of different probe liquids on the same solid surface

**DOI:** 10.1007/s00396-012-2777-9

**Published:** 2012-09-07

**Authors:** Emil Chibowski, Malgorzata Jurak

**Affiliations:** Department of Physical Chemistry-Interfacial Phenomena, Faculty of Chemistry, Maria Curie-Sklodowska University, 20-031 Lublin, Poland

**Keywords:** Contact angles hysteresis, DPPC monolayers, Solid support

## Abstract

Advancing and receding contact angles of water, formamide and diiodomethane were measured on 1,2-dipalmitoyl-*sn*-glycero-3-phosphocholine (DPPC) layers deposited on three different solid supports—glass, mica and poly(methyl methacrylate). Up to five statistical monolayers were deposited on the surfaces by spreading DPPC solution. It was found that even on five statistical DPPC monolayers, the hysteresis of a given liquid depends on the kind of solid support. Also on the same solid support the contact angle hysteresis is different for each probe liquid used. The AFM images show that the heights of roughness of the DPPC films cannot be the primary cause of the observed hysteresis because the heights are too small to cause the observed hystereses. It is believed that the hysteresis is due to the liquid film present right behind the three-phase solid surface/liquid drop/gas (vapour) contact line and the presence of Derjaguin pressure. The value of contact angle hysteresis depends on both the solid surface and liquid properties as well as on intermolecular interactions between them.

## Introduction

Although contact angle hysteresis phenomenon has been well known for several decades [1], its nature is not fully explained yet. Numerous papers have been published in which this phenomenon was investigated both experimentally and theoretically, and it would be impossible to cite all of them. The appearance of contact angle hysteresis was originally attributed to the surface roughness, its chemical heterogeneity and/or surface active impurities present in the liquid. Some general characteristic features of the hysteresis, among others, are discussed in Adamson and Gast’s monograph [2].

As is well known, practically on all real solid surfaces contact angle hysteresis is observed, and one may claim that all real surfaces possess some roughness and/or chemical imperfections of the surface structure on the molecular scale. The microscopic contact angle can vary across such surfaces, and the experimental macroscopic advancing and receding contact angles measured using a few microlitre droplet settled on the surface are averaged values of the microscopic angles. Various aspects concerning relations between microscopic and macroscopic contact angles, also in relation to contact angle hysteresis, were published by Decker et al. [3]. Among other findings, they concluded that “amount of roughness (at the most Ångstrom level) of the contact line does not correlate with the amount of hysteresis”, resulting from “dramatic changes in the advancing and receding contact angles” that occurred after UV/ozone treatment. Gaydos and Neumann [4] deduced that minimum patch size of the heterogeneous surface to produce contact angle hysteresis was about 1 μm. The effect of surface roughness on the hysteresis may depend not only on the roughness heights but also the roughness topology [5]. On the other hand, Krumpfer and McCarthy [6] stated that because the “advancing and receding events are not generally the reverse of one another… this leads to the expectation that most surfaces should exhibit contact angle hysteresis—even if they are not dirty, rough or chemically heterogeneous”. Indeed, even on smooth and low surface energy solids like Teflon [7, 8], self-assembled hexadecyltrichlorosilane monolayers deposited on glass slide or silicon [3], or polystyrene with fluorocarbon substituents [9], a considerable hysteresis of water and other liquids was observed. Lately, Krumpfer and McCarthy [6] discussed contact angle hysteresis appearing on hydrophobic and superhydrophobic surfaces concluding that it “is due to receding contact line pinning”, and the amount of hysteresis depends on “the sign of curvature of the tops of posts”. Simultaneously, the authors describe procedures for obtaining smooth surfaces with the hysteresis ranging within 0.5–1° which can be obtained, for example, by covering the silicon surface with poly(dimethylsiloxane) through the process of silanization.

Different origins of hysteresis, which appears even on molecularly smooth solid surfaces, have been recently presented by Starov and Velarde [10]. They considered thermodynamic equilibrium of the system: solid surface/liquid drop/gas and the presence of Derjaguin pressure behind the three-phase contact line. They concluded that although in some systems contact angle hysteresis was due to surface roughness and its mechanical or chemical heterogeneity, the“hysteresis could be found even on homogeneous perfectly flat surfaces as a consequence of the peculiar shape of the Derjaguin isotherm in the partial wetting case”. Moreover, they also found that static advancing contact angle is not affected by the solid surface roughness if its height is less than 10–30 nm. Also, Diaz et al. [11] discussed the so-called intrinsic hysteresis appearing during measurement of static contact angle in which an adsorbed film at the three-phase contact line is considered. Earlier Chibowski [12, 13] assumed the presence of the liquid film behind the drop after the three-phase line has retreated and derived an equation for calculation of the apparent surface free energy from the contact angle hysteresis. It should be mentioned that in most published papers, the contact angle hysteresis mostly deals with water sessile drops on different solid substrates, from partially hydrophilic up to superhydrophobic, and relatively small number of papers have been published where contact angle hysteresis of liquids other than water is reported [14–16].

Therefore, the purpose of this study was to investigate contact angle hysteresis of water, formamide and diiodomethane on phospholipid 1,2-dipalmitoyl-*sn*-glycero-3-phosphocholine (DPPC) layers deposited on three different solid supports, i.e. glass, mica and poly(methyl methacrylate) (PMMA). Up to five statistical monolayers were deposited on each solid surface. It seemed to us interesting to learn whether and how much the kind of solid support, covered with the same amount of DPPC monolayers, affected the contact angle and its hysteresis. Another question was whether on the same surface the amount of hysteresis was different depending on the liquid used. For the bare solid surfaces and surfaces with two statistical DPPC monolayers deposited the AFM images were recorded.

## Experimental

### Materials

DPPC (semi-synthetic, 99 %) was purchased from Sigma and used without further purification. The probe liquids employed for contact angle measurements were: water from Milli-Q Plus system (resistivity, 18.2 MΩ cm), formamide (UCB Co., Belgium, >99 %) and diiodomethane (POCh S.A, Gliwice, Poland, p.a.).

### Methods

Before deposition of DPPC, the surfaces of microscope glass slides (20 × 26 mm), mica (Continental Trade, Warsaw, Poland) plates (8 × 16 mm) and PMMA (Plastic-Group, Lublin, Poland) plates (20 × 20 mm) were carefully prepared. The details of surfaces pretreatment were published earlier [17]. The successive DPPC layers were obtained by pouring from a microsyringe appropriate volume of its aqueous solution, or in the case of PMMA methanolic solution, on the solid support surfaces. The volume of the solution corresponded to one statistical monolayer of DPPC. It was calculated taking 0.57 nm^2^ area for a DPPC molecule and the geometric surface of the solid plate. The consecutive layer was deposited on the previous one by depositing the same volume of the solution on the surface covered with dried DPPC layer(s). This procedure was repeated up to deposition of five monolayers. The details of the layers preparation by spreading from solution were described elsewhere [17].

The contact angles were measured at room temperature (20 ± 1 °C) using a GBX Contact Angle Meter (France) equipped with a video-camera system and computer software for the contact angle calculation from the shape of the sessile droplet. The advancing contact angle was measured after depositing 3-μL droplet on the surface. Then, a 1-μL volume was sucked into the syringe from the droplet, and receding contact angle was read out. The readings were taken both on the left and right sides of the 2D droplet profile for all three test liquids and on each statistical monolayer of DPPC deposited on the solid supports. Three series of the contact angle measurements were conducted on each support using the three liquids. In each series and for each liquid, the contact angles were measured for two to three droplets. Thus, the arithmetic mean value of the contact angle of a particular liquid was calculated from about 12–18 readings.

The topography of bare solid surfaces used as the supports and those with two statistical DPPC monolayers deposited was imaged with an Atomic Force Microscope Nanoscope III (Veeco, USA) equipped with standard silicon or silicon nitride tips. All images were recorded in contact or tapping mode. The AFM images were analysed using WSxM program (Version Develop 8.0 Scanning Probe Microscope software) [18].

## Results and discussion

**Fig. 1:**
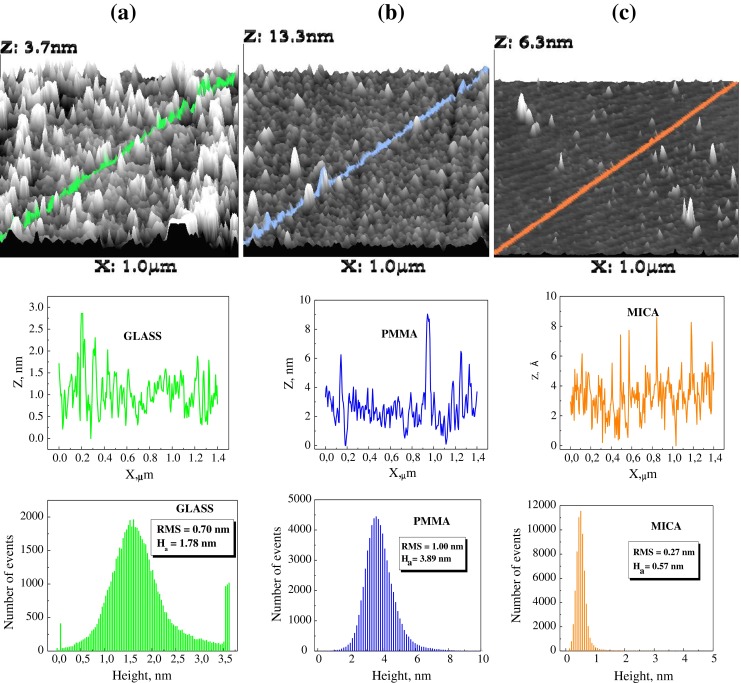
3D AFM images of the bare surfaces of glass (**a**), PMMA (**b**) and mica (**c**), the roughness along the *marked line*, and the roughness of total surface shown from *top to bottom*, respectively. Also, RMS and average height values, *H*_a_, are given

**Fig. 2:**
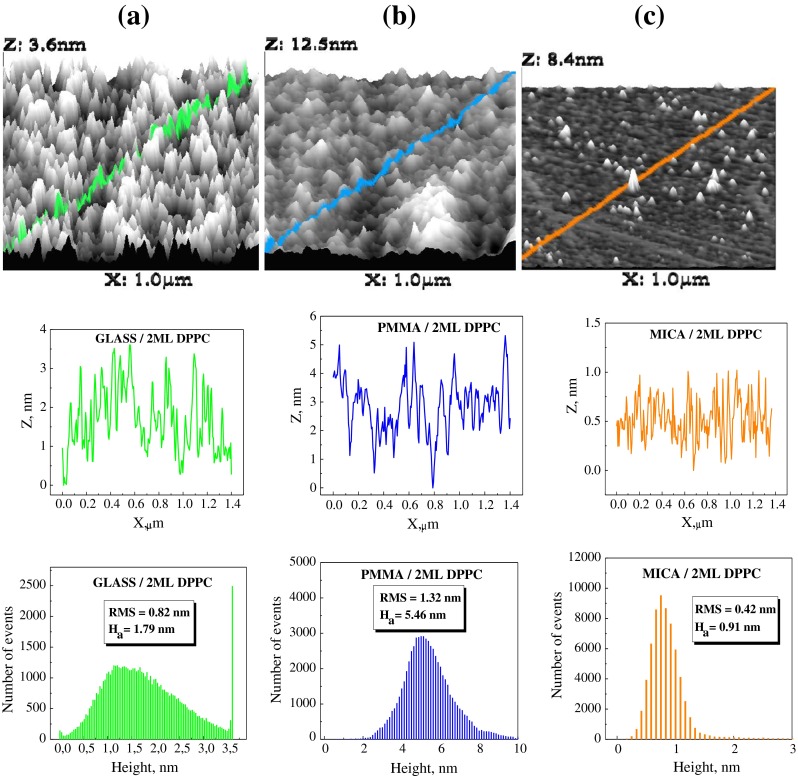
3D AFM images of two statistical DPPC monolayers deposited on the surfaces of glass (**a**), PMMA (**b**) and mica (**c**), the layer roughness along the *marked line*, and roughness of the total surfaces from *top to bottom* are shown, respectively. Also, RMS and average height values, *H*_a_, are given

The 3D AFM images of the bare solid substrates and their roughnesses are shown in Fig. 1. The most flat surface is that of mica. The average roughness of its surface is 0.57 nm. Also, average surface roughnesses of glass and PMMA are small, 1.78 and 3.89 nm, respectively. These values are much below those (10–30 nm) discussed by Starov and Velarde that would affect static advancing contact angle [10]. Therefore, the contact angle hysteresis, if appears on these surfaces, can be considered as resulting from different origin than the surfaces roughness. This is as an example, in Fig. 2 are presented 3D AFM images of the two statistical DPPC monolayers deposited on the solid surfaces shown in Fig. 1. Again, the average roughness of the layers deposited on mica is the smallest one, and that on glass is the same as that of bare glass surface. Also on PMMA surface, the roughness is not much larger than that on its bare surface (compare Figs. 1 and 2). In the case of the DPPC layers, the distribution of the roughness is broader than that of the appropriate bare support surface. Anyway, it can be concluded that the layers roughness should not be a direct cause of the contact angle hysteresis if it appears.

The surface tension and its components of the probe liquids: water, formamide and diiodomethane [19], their boiling point, vapour pressure, the molecule volume and the area per molecule are listed in Table 1. The two liquids, water and formamide, are polar and diiodomethane is apolar liquid. The liquids evidently differ in their vapour pressure at room temperature and the molecular size (volume), as well as in the nature and strength of the interactions.

**Table 1:** Surface tension and its components of the probe liquids [19] used for contact angle measurements in milli-Newton per meter, and some other parameters characterizing the liquids

Probe liquid	*γ*_L_	*γ*_L_^LW^	*γ*_L_^+^	*γ*_L_^–^	*γ*_L_^AB^	Boiling point °C	Vapour pressure at 20 °C mmHg	Volume per molecule, nm^3^	Area per molecule, nm^2^
Water, H_2_O	72.8	21.8	25.5	25.5	51.0	100	17.5	0.030	0.059
Formamide, HCONH_2_	58.0	39.0	2.28	39.6	19.0	210	0.08	0.066	0.197^a^
Diiodomethane, CH_2_I_2_	50.8	50.8	0	0	0	182	0.82	0.134	0.215

*γ*_*L*_ liquid surface tension, *γ*_*L*_^*LW*^ liquid Lfshitz–van der Waals component, *γ*_*L*_^*+*^ liquid electron acceptor component, *γ*_*L*_^*–*^ liquid electron donor component

^a^Calculated assuming spherical shape of the molecule

Before discussing the contact angles presented in this paper, some problems dealing with the interpretation of experimentally measured contact angles should be briefly mentioned. A detailed discussion on this issue can be found in a paper published by Marmur [20], although he has introduced many forms of contact angles, like: *geometric, ideal, Young, actual, apparent, advancing, receding, most stable and dynamic,* and one may become a bit confused as to what contact angle is actually measured in a given solid/liquid drop/gas system. On the other hand, a careful reading of this paper helps understanding the problems encountered in the interpretation of the experimental contact angles. Anyway, the experimental contact angle is always a *macroscopic* quantity, and its value can be an average of many *microscopic* contact angles that may appear around the droplet perimeter on a rough solid surface. However, as was mentioned above, it is the case of a solid surface having roughness of micrometer size [3, 10, 20]. Nevertheless, in practical liquid drop/solid surface systems, the macroscopic contact angle is *an apparent* *contact angle*. If the surface is micrometrically rough, then the ratio of the droplet size to the roughness size should be two to three orders of magnitude larger in order to measure meaningful apparent contact angles [20]. In the systems investigated in this paper, the roughness of the surfaces is only a few nanometers; therefore, the droplet of 3 μl volume completely fulfills the condition of the above-mentioned ratio, and because during depositing the droplet from automatic deposition system on the solid surface its volume is slowly increased up to 3 μl, we consider thus measured contact angle as an apparent advancing contact angle, which is probably close to the maximum advancing contact angle [20]. Then, after sucking 1 μl volume of the liquid from the settled droplet into the syringe, the three-phase contact line has retreated, and the measured contact angle is the apparent receding contact angle, which again can be considered as that close to the minimum receding contact angle. It is believed that behind the droplet a liquid film is present [10, 11, 13, 15, 21]. Both thus measured contact angles are equilibrium ones, but they are not Young’s contact angles [20].In consequence, the observed hysteresis of a given probe liquid contact angle can be considered as the difference between the apparent contact angle of the droplet surrounded by bare solid surface and the apparent receding contact angle of the droplet behind which some film of the liquid is present.

**Fig. 3:**
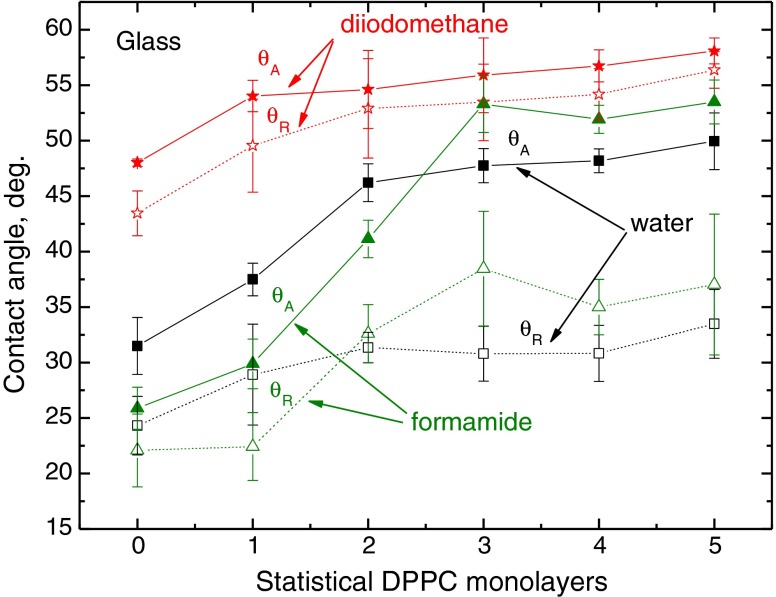
Advancing and receding contact angles of water, formamide and diiodomethane on DPPC monolayers deposited on glass

**Fig. 4:**
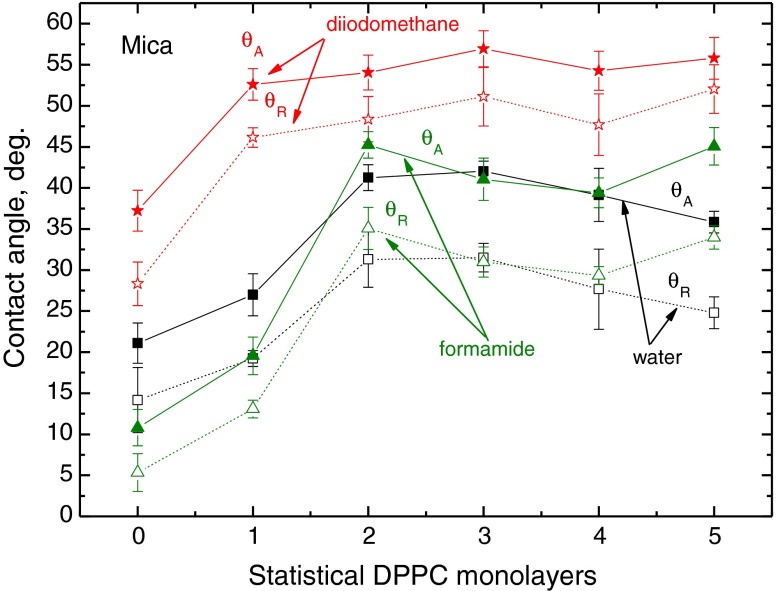
Advancing and receding contact angles of water, formamide and diiodomethane on DPPC monolayers deposited on mica

**Fig. 5:**
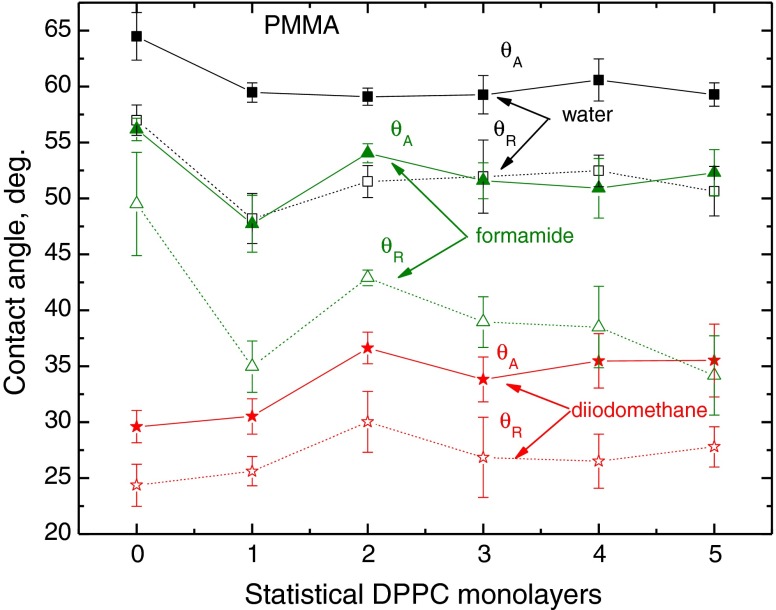
Advancing and receding contact angles of water, formamide and diiodomethane on DPPC monolayers deposited on PMMA

The advancing and receding contact angles of these liquids on the DPPC layers deposited on glass, mica and PMMA are plotted in Figs. 3, 4 and 5. The first general conclusion that can be drawn by analysing these results is that while on strongly polar solid substrates (glass and mica), the largest contact angles (both advancing and receding) were measured for diiodomethane droplets (Figs. 3 and 4), and on weakly polar PMMA substrate, the contact angles of diiodomethane are the lowest among these three probe liquids (Fig. 5). It can be also easily noticed that generally the diiodomethane contact angle hysteresis is the smallest. The second general observation is that the reproducibility of the measured contact angles is relatively good, especially that of the advancing ones. It is a well-known fact that reproducibility of receding contact angle is generally worse than that of advancing, both measured on the same surface. The third observation is that larger changes in the contact angles occur with the increasing number of DPPC layers deposited on higher energy surfaces of glass and mica than on those deposited on weakly polar PMMA, whose surface possesses only some electron donor interactions (*γ*_s_^−^ = 10–20 mJ/m^2^) [21–23]. Finally, the greatest changes in the probe liquids contact angles occur on first two to three statistical monolayers when compared to those on the bare substrate surface.

**Fig. 6:**
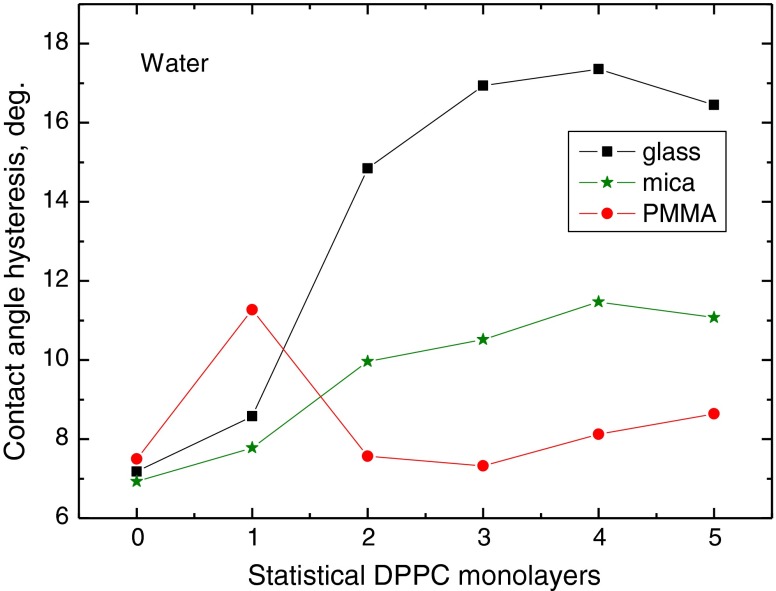
Contact angle hysteresis of water on DPPC monolayers deposited on glass, mica and PMMA

**Fig. 7:**
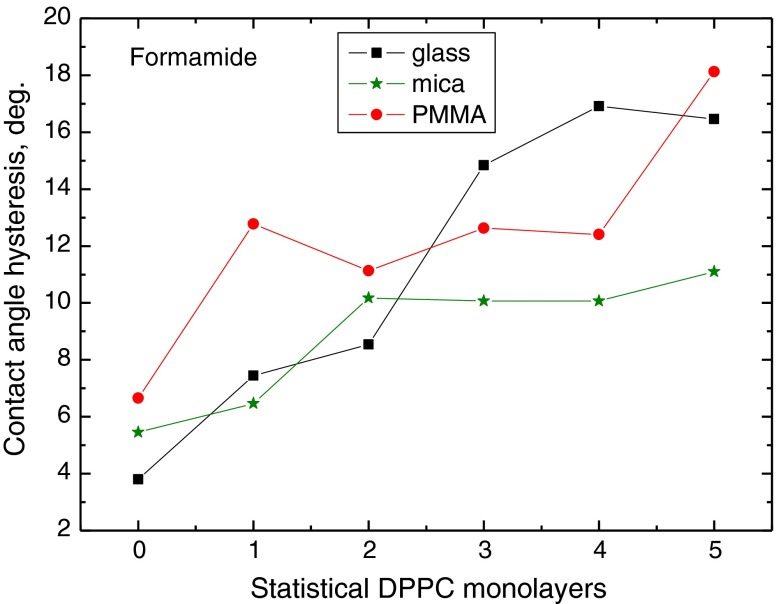
Contact angle hysteresis of formamide on DPPC monolayers deposited on glass, mica and PMMA

**Fig. 8:**
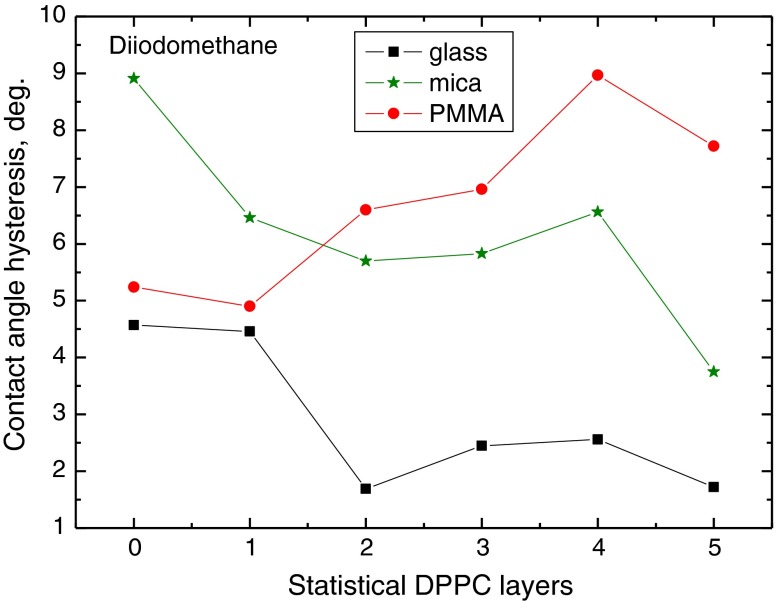
Contact angle hysteresis of diiodomethane on DPPC monolayers deposited on glass, mica and PMMA

To better depict the effect of the substrate surface properties on the contact angle hysteresis, in Figs. 6, 7, and 8 are plotted the contact angle hysteresis values for water, formamide and diiodomethane on the DPPC layers deposited on glass, mica and PMMA. Water contact angles hysteresis values are plotted in Fig. 6. It increases sharply with increasing number of monolayers on glass (from 7° to 17°), much less on the monolayers deposited on mica (from 7° to 11°), and it does not change much on the layers on PMMA (6–8°), except that on one statistical DPPC monolayer (11°), where it is larger than on glass and mica. These results evidently show that contact angle hysteresis of highly polar water depends on the solid substrate surface properties, even though up to five DPPC monolayers were deposited. It may be postulated that this must be due to differences in the layers structures and DPPC molecules orientation, which to some extent is confirmed by AFM images too (Fig. 2). Moreover, some restructuring of the DPPC layer structure upon prolonged contact with water can also occur [24, 25]. However, this is not so evident in the case of formamide contact angles measured on the same DPPC layers, which is also a polar liquid whose hysteresis results are shown in Fig. 7. In this case, generally the hysteresis increases with the DPPC layer thickness on these three solid substrates, and it practically does not change on three to five layers on mica (10–11°). However, no clear relationship can be found with the kind of substrate. In Fig. 8 are plotted the contact angle hysteresis values of apolar diiodomethane droplets on the DPPC layers. The hysteresis changes for this liquid show a different trend than those for water and diiodomethane. On the layers deposited on glass and mica, the hysteresis decreases with increasing layers thickness, and it increases on the layers present on PMMA surface. In fact, the diiodomethane contact angle hysteresis in all cases is below 9° ranges between 2° and 9°.

To summarize the above discussed results, it is evident that contact angle hysteresis on the same DPPC film, but deposited on different solid substrates, differs significantly for highly polar water and practically apolar diiodomethane. On the other hand, this is not the case for the contact angle hysteresis of formamide (Fig. 7). As the heights of the roughness are not the principal cause of the observed hysteresis [10], it must be due to some differences in structures of the layers, and the contact angle hysteresis of a given probe liquid depends not only on the liquid nature but also on the DPPC molecule/solid surface interactions which reflect in the orientation of the DPPC molecules, i.e. with their polar head or hydrophobic tails outward. On strongly polar glass and mica surfaces, more hydrophobic tails should be directed outward than in the case of weakly polar PMMA surface, on which they may be less ordered. The AFM images (Fig. 2) and the contact angles (Figs. 3, 4 and 5) show that the DPPC layers might not be tightly packed and uniform, if deposited in the above described way, and the packing, organization and orientation of the molecules in the film are those of a gel state. Hence, water can penetrate the film and interact also with the bare support surface and, therefore, the contact angles are relatively low on the films deposited on glass and mica surfaces, and much higher on the films on PMMA surface.

The increase in contact angle hysteresis also points that the probe liquid droplet penetrates deeper into the phospholipid layer structure during the receding of the three-phase contact line. Hence, the differences in the values of contact angles and their hystereses reflect different strengths of the solid/liquid interactions.

However, the nature of the liquid molecule is of great importance too. Especially large differences in the contact angle hysteresis are observed on the two to five statistical monolayer-thick layers deposited on different substrates (Figs. 6, 7 and 8). Water is the smallest molecule among these probe liquids (Table 1), and according to van Oss et al. [19], it shows strong electron donor *γ*_L_^−^ and electron acceptor *γ*_L_^+^ interactions, while formamide possesses only strong *γ*_L_^−^ parameter. Diiodomethane molecules interact almost entirely by London dispersion forces. The access of the probe liquid molecules to the polar head and apolar chains of DPPC molecules is reflected in the measured contact angles. This access seems to be more important in the case of receding contact angles measured after the three-phase contact line of the droplet has retreated, and the liquid may or may not penetrate into the DPPC layers, and thus, a liquid film is left behind the drop.

Some additional information can be obtained from the calculated surface free energies using the contact angle hysteresis approach, Eq. (1) [12, 13].

1 $$ \gamma_{\text{s}}^{\text{tot}} = \frac{{{\gamma_{\text{L}}}{{\left( {1 + \cos {\theta_a}} \right)}^2}}}{{\left( {2 + \cos {\theta_{\text{r}}} + \cos {\theta_{\text{a}}}} \right)}} $$

**Fig. 9:**
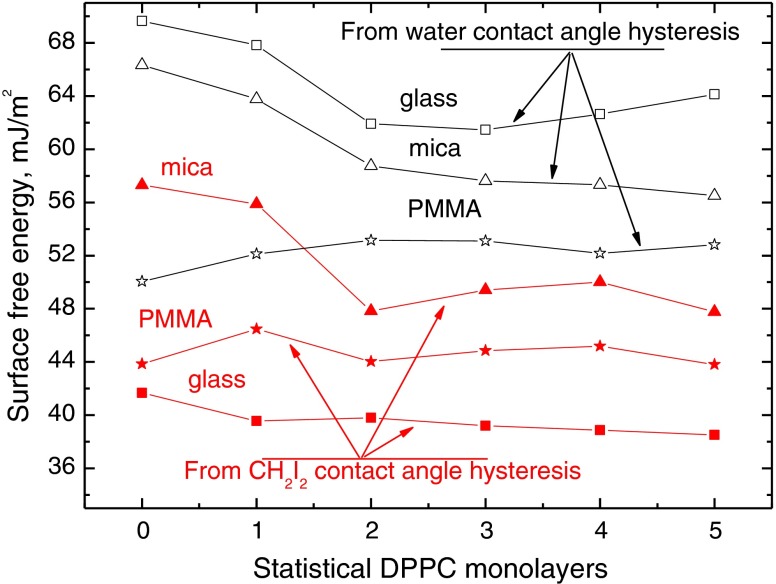
Apparent surface free energy of DPPC monolayers deposited on glass, mica and PMMA calculated from contact angle hysteresis of water and diiodomethane (CH_2_I_2_)

Thus, calculated values are apparent ones, as in fact macroscopic contact angles are too, and this model has been discussed elsewhere [12, 13]. Despite the values are apparent ones, they provide information about the surface free energy changes taking place with increasing thickness of the layers and depending on the nature of the liquid used, i.e. polar or apolar. Thus, calculated values from contact angle hysteresis of water and diiodomethane for DPPC layers deposited on the three solid supports are plotted in Fig. 9. In the case of layers deposited on glass and PMMA, the apparent surface free energy values determined from diiodomethane contact angles are practically constant on all five layers, and they differ only slightly from those of bare solids, respectively, despite some changes in the advancing and receding contact angles and their hysteresis that occurs (Figs. 3, 5, 6 and 8). This means that the London dispersion interactions are similar. However, the surface free energy calculated from diiodomethane contact angles for DPPC layers deposited on mica decreases significantly, about 9 mJ/m^2^ on the two to five monolayer-thick layers (Fig. 9), in comparison to the bare surface. This means that much stronger dispersion interactions are present on the bare mica surface than on DPPC monolayers, where the dispersion energy of the hydrocarbon tails seems to be weaker. Thus, the apparent surface free energy values determined from water contact angles hysteresis are higher than those determined from diiodomethane contact angles (Fig. 9). The smallest changes appear in the case of DPPC layers deposited on PMMA whose surface shows relatively weak electron donor *γ*_S_^−^ and no electron acceptor *γ*_S_^+^ interactions [21–23]. This suggests that polar heads of DPPC molecules are less accessible than on the two other substrates. The DPPC layers’ energy changes on glass and mica determined from water contact angles run almost parallel to each other, and they are a little bigger on glass. The greatest energy decrease occurs on first two monolayers, and then the changes are small. Thus, the interactions of polar water molecules are weaker on the layers than on the bare surface of glass and mica, on which probably stronger hydrogen bonding can be formed.

## Conclusions

Contact angle hysteresis depends on both the solid and liquid properties. On the studied DPPC layers deposited on three different solid supports, the contact angle hysteresis is larger for polar liquids (water and formamide) than apolar diiodomethane. The surface tension and its components of the probe liquids are different, as well as size of their molecules and the vapour pressure at room temperature. The hysteresis is larger on the DPPC layers deposited on the surfaces having strong polar interactions, i.e. glass and mica, than on weakly polar PMMA (The differences in hysteresis amount appear even on five statistical monolayers of DPPC). For water and formamide, the hysteresis increases with the layer thickness increase, but for diiodomethane, it increases only on the layers deposited on PMMA and decreases on the layers deposited on glass and mica. Such behaviour clearly indicates that interrelation between solid support and liquid properties is decisive for the contact angle and its hysteresis on the same kind of layer, here DPPC. It is believed that in the studied systems, the contact angle hysteresis does not result from the surface (film) roughness, which is only a few nanometers high, but that it is due to the liquid film presence behind the drop (Derjaguin pressure) in the receded state of the droplet. Additional information can be obtained by calculation of the apparent surface free energy from the contact angle hysteresis, where both the advancing and receding contact angles are taken into account. Thus, the obtained results give better insight into liquid/solid surface interactions which depend both on the liquid and the surface properties.
